# Assessment of angiogenesis by CD105 antigen in epithelial salivary gland neoplasms with diverse metastatic behavior

**DOI:** 10.1186/1471-2407-9-391

**Published:** 2009-11-04

**Authors:** Sergio V Cardoso, Kelen Christine N Souza, Paulo R Faria, Ana Lucia A Eisenberg, Fernando L Dias, Adriano M Loyola

**Affiliations:** 1 Pathology Area, School of Dentistry, Federal University of Uberlandia, Brazil; 2 Pathology Post-Graduation Programme, Federal University of Triangulo Mineiro, Brazil; 3 Biological Sciences Institute, Federal University of Uberlandia, Brazil; 4 Pathology Division, National Cancer Institute, Brazil; 5 Head and Neck Surgery Section, National Cancer Institute, Brazil

## Abstract

**Background:**

Information on the biology of metastasis development in salivary gland tumors is scarce. Since angiogenesis seems associated with this phenomenon in other tumors, we sought to compare salivary gland tumors with diverse metastatic behavior in order to improve the knowledge and management of these lesions.

**Methods:**

Samples from the most important salivary gland tumors were segregated according to its metastatic behavior and submitted to routine immunohistochemistry to identify vessels positive for CD105 expression. Frequency of positive cases and intratumoral microvessel density (IMD) was compared among the group of lesions.

**Results:**

CD105 positive vessels were absent in normal salivary gland tissue, were rare in pleomorphic adenomas and adenoid cystic carcinomas (ACC), more common in polymorphous low-grade adenocarcinomas and highest in mucoepidermoid carcinomas. Only ACC with such feature were metastatic. IMD was higher in malignant rather than benign tumors.

**Conclusion:**

Immunostaining of CD105 in salivary gland tumors implies participation of angiogenesis in the development of malignant lesions, as well as some role for myoepithelial cells in the control of new vessel formation. In addition, suggest that ACC with positive CD105 vessels are at higher risk for metastasis.

## Background

Malignant salivary gland tumors constitute a major challenge in head and neck oncology because of its frequency, varied histological typing, difficult surgical approach, and poor response to other therapies. It is further complicated by the absence of clear parameters to preview biological behavior, in particular to predict development of metastasis. In this sense, there is few information regarding pathways implicated in tumor dissemination of salivary gland cancer.

Tumor growth is limited by the balance between neoplastic demand for oxygen and nutrients and diffusion from preexisting surrounding vascularization, and therefore formation of new blood vessels is an essential step in progression of cancer [1]. In general, this process is named angiogenesis, requires release of activating mediators as well as suppression of inhibitory mechanisms, and may evolve through several mechanisms, such as sprouting, intussusception, recruitment, cooption, and mimicry [2]. Both angiogenesis and neoplastic invasion and metastasis are associated with increasing vascular surface, remodeling of extracellular matrix, and secretion of growth factors [3].

In situ quantification of microvessel density by immunohistochemistry is a usual form to assess angiogenesis for different types of neoplasia. However, inconsistencies and contradictions have been pointed out, in particular because of the use of endothelial markers which are unable to distinguish true angiogenic endothelial activity [4-6]. CD105, also known as endoglin, is a receptor for TGFβ signaling, and play an important role in angiogenesis and fibrogenesis [7,8]. It is essential for endothelial cell proliferation, thus promoting the activation phase of angiogenesis [9]. More important, CD105 expression is a prominent feature of newly formed tumor vessels but minimally expressed in quiescent preexisting ones [7,10]. It is also a prognostic marker for squamous cell carcinoma of the head and neck [11,12].

Finally, there is few information translating the relationship between angiogenesis and dissemination of salivary gland tumors to clinical-driven parameters. In this sense, this work was conducted to investigate whether lesions with diverse clinical course also differ in their neovascular content.

## Methods

### Tissue samples and patients characteristics

The use of archived human tissues, as well as the entire research protocol, was reviewed and approved by the Institutional Review Board of the Brazilian National Institute of Cancer (reference 42/04). Histological samples and follow up data were obtained from the Division of Pathology and from the Head and Neck Service, respectively, of the Brazilian National Cancer Institute, Rio de Janeiro, Brazil. It was initially gathered cases of epithelial salivary gland neoplasms that were surgically resected as the first therapeutic intervention in the period between 1998 and 2004. Further selection of those patients with specific histological types of interest, with the most characteristic features for each type as reviewed by an expert pathologist (A. M. L.) [13], and minimum follow-up of five years or up to identification of metastasis, resulted in a final series of 139 cases, comprising pleomorphic adenomas (PA), adenoid cystic carcinomas (ACC), polymorphous low-grade adenocarcinomas (PLGA), and mucoepidermoid carcinomas (MEC). Non-neoplastic salivary gland tissue at the periphery of the samples was also screened.

### Immunohistochemistry

CD105 expression was assessed in 3 μm thick tissue sections cut from formalin-fixed, paraffin-embedded specimens. Antigen retrieval was performed in deparaffinized, rehydrated samples with 1 mM EDTA (pH 8.0) in microwave environment (3 × 5 min). After washing in distilled water and TRIS-HCl buffer (0.5 M, pH 7.4), the slides were incubated in 3% hydrogen peroxide (20 min), once again washed, and incubated with endogenous avidin and biotin-blocking solution (purchased from Dako, Carpinteria, CA, USA) for 20 minutes each. Slides were rinsed with water and buffer, and then primary mouse antibody (clone SN6h, purchased from Dako), titrated at 1:100, was applied for 19 hours at 4°C. Amplification of the reaction was performed with secondary biotynilated anti-mouse antibody (Vector Labs, Burlingame, CA) and streptavidin-biotin-peroxidase (Dako) for 30 minutes each. After extensive washing, reaction was revealed with 3,3'-diaminobenzidine hydrochloride (Sigma, St. Louis, MI) and 0.02% peroxide hydrogen, followed by counterstaining with Mayer's hematoxylin. Omission of the primary antibody was employed as negative control, while large B-cell lymphoma and tonsil tissue were used as positive controls.

Intratumoral microvessel density (IMD) was quantitated according to a recent consensus statement [14]. Briefly, in an optical microscope (E400, Nikon Instruments, Melville, NY) hot-spot areas for CD105 expression in discrete blood vessels were initially identified by scanning the entire tumor at low power (× 50 and × 125). The number of CD105 highlighted vessels in 10 of these areas was then counted in high power magnification (× 500).

### Statistics

Possible associations between metastatic behavior and positivity for CD105 were tested by chi-square or Fisher's exact tests. Non-parametric tests were employed to evaluate differences in IMD between benign and malignant tumors, primary non-metastasizing and metastasizing malignant lesions (Mann-Whitney test), and among different histological types of neoplasms (Kruskal-Wallis with Dunn's post hoc test).

## Results

**Figure 1:**
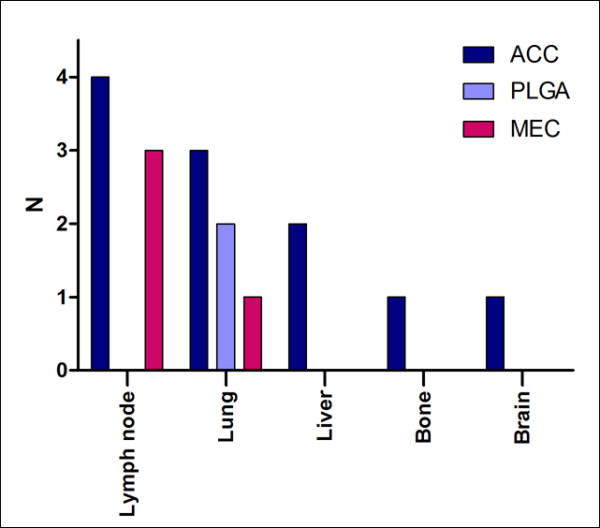
**Distribution of metastasizing tumors, according to histological type and site of metastatic implants**.

Most of the patients were women (60.4%), ranging from 6 to 86 years of age at the time of admission, as shown in Table 1. For malignant tumors, median follow-up time was 44.1 months, metastatic dissemination was observed in 16 cases (14.7% of these lesions), and three patients (two ACC and one MEC) had two organs affected (Figure 1).

**Table 1:** Clinical and demographic data

	Sex	Age
	
Class or type of tumor	(male:female)	(years, mean ± SD)
**Benign (pleomorphic adenoma)**	1:1.5	52.0 ± 13.8
**Malignant**	1:1.5	50.3 ± 17.1
**Malignant, non-metastasizing**	1:1.9	49.6 ± 17
**Malignant, metastasizing**	1:0.4	54.5 ± 17.6
**Adenoid cystic carcinoma**	1:1.8	51.2 ± 16.6
**Polymorphous low-grade adenocarcinoma**	1:1.1	61.1 ± 13.3
**Mucoepidermoid carcinoma**	1:1.5	44.8 ± 17.9

**Figure 2:**
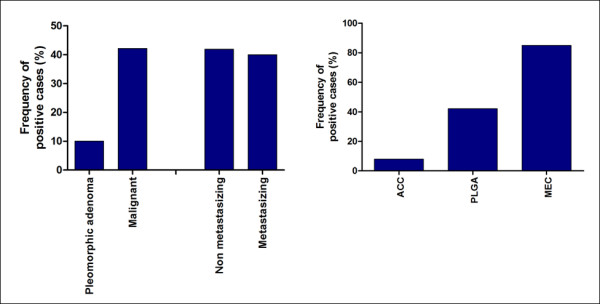
**Frequency of cases with positive staining for CD105, according to: A. biological behavior; and B. histological type**.

**Figure 3:**
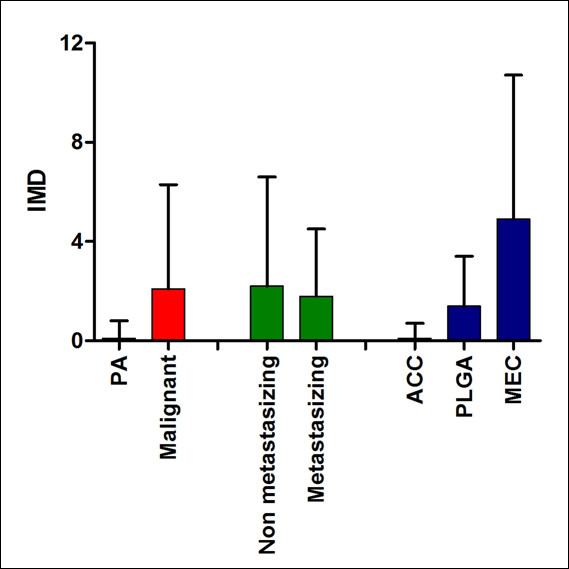
**Intratumoral microvessel density (IMD), evaluated by CD105 staining, according to biological behavior and histological type**.

CD105 positive vessels were absent in normal salivary gland tissue in the vicinity of the tumors (present in 83 samples). In tumors, staining was observed as cytoplasmic brown deposits in endothelial cells of capillary vessels. Frequency of CD105 positivity was significantly higher in malignant (42.2%) rather than pleomorphic adenomas (with 10.0%; *p *= 0.001, Fisher's exact test), but it was very similar between non-metastasizing and metastasizing primary malignant tumors (Figure 2A). Among malignant neoplasms, there was graded frequency of positivity for CD105, with MEC being the highest (85.0%), followed by PLGA (42.1%), and ACC (with 8.0%; *p *< 0.0001, chi-square test, Figure 2B). IMD was also elevated in malignant neoplasms compared to benign tumors (*p *= 0.004, Mann-Whitney U test), similar in non metastasizing and metastasizing malignancies, and significantly higher in MEC than ACC or PLGA (*p *< 0.0001, Kruskal-Wallis with Dunn's post-hoc test), as show in Figure 3. Intensity of staining was also much stronger in MEC.

**Figure 4:**
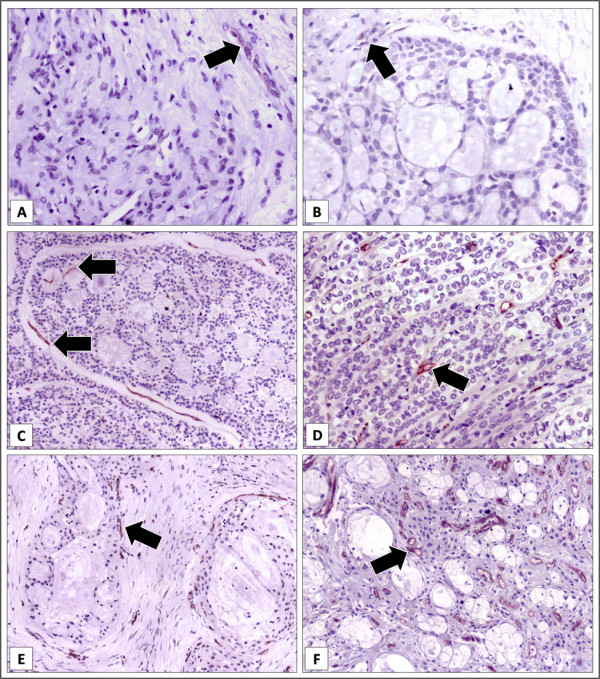
**Immunohistochemical staining of CD105 in salivary gland tumors (streptavidin-biotin-peroxidase technique, original magnification = × 250)**. A. Pleomorphic adenoma, depicting very weak staining (arrow). B. Only one primary non metastasizing adenoid cystic carcinoma was positive for CD105 staining, of weak intensity (arrow). C. Adenoid cystic carcinoma, primary metastasizing lesion, depicting clear and strong positivity around and within tumoral nests (arrows). D. Polymorphous low-grade adenocarcinoma, primary non-metastasizing lesion, with strongly positive vessels (arrow) permeating tumoral strands. E. Mucoepidermoid carcinoma, primary non-metastasizing lesion, with strongly positive vessels (arrows) around tumoral nests. F. Mucoepidermoid carcinoma, primary metastasizing lesion, evidencing numerous and strongly positive vessels (arrows) intermingling tumoral parenchima.

For each histological type, positivity was almost restricted to metastasizing ACC (in three out of ten cases; only one non-metastasizing case depicted very few positive blood vessels) and non-metastasizing PLGA (in eight out of seventeen cases), while discretely increased IMD was observed for metastasizing MEC compared to non-metastasizing cases (Table 2). Representative images of CD105 positivity according to metastatic behavior of the lesions is presented in Figure 4. For the very few PA with CD105 positive vessels, it was rare, of weak intensity, and only focally observed in apparent fibrous septa. For ACC, positivity was of weak to moderate intensity, and it was mostly observed surrounding tumoral nests. For PLGA, CD105 positivity was predominantly weak to moderate, and it was mostly found at the periphery of the lesions, but eventually permeating tumoral parenchyma. Only for MEC, strongly CD105 positive vessels were observed in most of the lesions, permeating through or surrounding tumoral nests.

**Table 2:** CD105 immunohistochemical localization in primary salivary gland tumors (IMD = intratumoral microvessel density) [14].

		CD105 positivity	
		
Type and metastatic status	*N*	Frequency	IMD (mean ± SD)
**Adenoid cystic carcinoma**			
Primary, non-metastasizing	41	2.4%	0
Primary, metastasizing	10	30.0%	0.7 (± 1.3)
**Polymorphous low-grade adenocarcinoma**			
Primary, non-metastasizing	17	47.1%	1.6 (± 2.0)
Primary, metastasizing	2	0	0
**Mucoepidermoid carcinoma**			
Primary, non-metastasizing	36	83.3%	4.8 (± 6.0)
Primary, metastasizing	4	100%	6.2 (± 2.1)

## Discussion

Metastasis is an important cause of death for patients with cancer and there is evidence pointing out for a role of angiogenesis in the dissemination of malignant tumors [15]. In an effort to investigate possible association between formation of new blood vessels and development of metastasis in salivary gland tumors, the present work investigated the most frequent of these lesions (PA) as a conceptual standard for non-metastasizing disease, as well as three of the most important malignant neoplasms for which metastasis is possible, but less (PLGA) or more (ACC, MEC) frequent [16,17]. These lesions also diverge regarding to differentiation and myoepithelial content [18]. Another considerably common salivary gland cancer, adenocarcinoma NOS, was not included because of the prominent and characteristic morphologic and behavioral heterogeneities of this group of lesions. Work covering prognosis and new insights into biology of this rare lesion has been recently released [19]. Other aggressive tumors were not included because of its rarity and, in spite of one of the largest number of primary samples in the literature, it was not possible to obtain a representative sample of metastatic implants because many were only detected by imaging exams in organs such as lungs, bones, liver, or brain. In addition, interpretation of the present data is limited by the acknowledgement that an overall five years follow-up time is too short to fully assess metastatic behavior for ACC, since late distant manifestations is a prominent finding in this disease [13,17,18].

CD105 expression has been demonstrated almost exclusively in venous and arterial vessels surrounding and permeating tumors [10]. It also has been detected in non-neoplastic tissues with increased angiogenic activity, such as embryonic development and wound healing [2,4]. Greater accuracy (mainly specificity) of CD105 in demonstrating new blood vessels compared with other molecules such as CD34 and factor VIII is clear in the literature [20-22]. As expected, in the present work CD105-positive vessels were absent in normal salivary gland. Even almost half of the tumors did not present such finding, corroborating the specificity of CD105 in demonstrating newly formed blood vessels, but it also evidences that neoplastic development does not always require prominent angiogenesis. Costa et al. [23] observed discrepancy between proliferation of salivary gland tumor cells and closeness to vessels stained by CD34, suggesting that possibly tumors such as ACC maintain metabolic activity via an oxygen-independent process. Anyway, there was a noticeable increase in the mean IMD from benign to malignant lesions, therefore indicating that vascularization contributes to or reflects aggressiveness in salivary gland tumors. Increased demand for blood supply because of accelerated growth or acquired ability of tumor cells to produce angiogenic factors could explain this finding [2].

In the present work, mean IMD assessed by CD105 was not significantly different between the entire samples of malignant tumors that did or not metastasized, and even all of the PLGA and more than half of the ACC that metastasized did not present CD105 positive vessels, suggesting that angiogenesis is neither an absolute determinant nor required for acquisition of metastatic phenotype in these salivary gland tumors. However, only metastasizing ACC and all of metastasizing MEC were positive for CD105, suggesting that angiogenesis would be a priming step for at least some of these lesions to disseminate, specially for ACC. Zhang and Peng [24] observed that ACC cell lineages with higher invasive and metastatic potential were also more efficient than other with low metastatic behavior in promoting migration and tube formation of endothelial cells *in vitro*.

Another interesting finding of the present work was the continuous increase of IMD from ACC to PLGA and then to MEC. It is not apparently correlated to the aggressiveness of these lesions, but their myoepithelial content may help to explain such difference. There is evidence that myoepithelial cells inhibit angiogenesis [25,26]. It could greatly impair angiogenesis in PA and ACC. This effect would be only partial in PLGA, where myoepithelial content is intermediate between ACC and MEC. The latter does not present myoepithelial differentiation, and therefore would not present their inhibitory effect. Similarly to the present results, Costa et al. [23] observed less frequent staining of vessels by CD105 in ACC than MEC. In addition, these authors observed almost identical mean IMD in non-metastasizing and metastasizing ACC, in the same way of the present work, but did not evaluate PLGA or metastasizing MEC.

## Conclusion

The results of the present work contribute to the knowledge on the biology of salivary gland tumors, since increased angiogenesis was observed in malignant tumors. Further, it was also higher in MEC than in PLGA and particularly than in ACC and PA, supporting a likely role of myoepithelial cells as regulators of the formation of new blood vessels. Diagnostic implications are also present, since those ACC with CD105 positive vessels seems to be at increased risk for metastasis.

## Competing interests

The authors declare that they have no competing interests.

## Authors' contributions

SVC: acquisition of data, analysis and interpretation of data; have been involved in drafting the manuscript; have given final approval of the version to be published.

KCNS: conception and design, acquisition of data, analysis and interpretation of data; have given final approval of the version to be published.

PRF: conception and design, analysis and interpretation of data; have been involved in revising the manuscript critically for important intellectual content; have given final approval of the version to be published.

ALAE: acquisition of data, analysis and interpretation of data; have been involved in revising the manuscript critically for important intellectual content; have given final approval of the version to be published.

FLD: conception and design, acquisition of data; have been involved in revising the manuscript critically for important intellectual content; have given final approval of the version to be published.

AML: conception and design, acquisition of data, analysis and interpretation of data; have been involved in revising the manuscript critically for important intellectual content; have given final approval of the version to be published.

## Pre-publication history

The pre-publication history for this paper can be accessed here:

http://www.biomedcentral.com/1471-2407/9/391/prepub

## References

[B1] Folkman J Mendelsohn J, Howley P, Israel A, Liotta LA Tumor angiogenesis The Molecular Basis of Cancer 1995 Philadelphia: W.B. Saunders Company 206 224

[B2] Hillen F Griffioen AW Tumour vascularization: sprouting angiogenesis and beyond Cancer Metastasis Rev 2007 26 3-4 489 502 10.1007/s10555-007-9094-7 17717633 PMC2797856

[B3] Lorusso G Rüegg C The tumor microenvironment and its contribution to tumor evolution toward metastasis Histochem Cell Biol 2008 130 6 1091 103 10.1007/s00418-008-0530-8 18987874

[B4] Du JR Jiang Y Zhang YM Fu H Vascular endothelial growth factor and microvascular density in esophageal and gastric carcinomas World J Gastroenterol 2003 9 7 1604 6 12854174 10.3748/wjg.v9.i7.1604 PMC4615515

[B5] Kumar S Ghellal A Li C Byrne G Haboubi N Wang JM Bundred N Breast carcinoma: vascular density determined using CD105 antibody correlates with tumor prognosis Cancer Res 1999 59 4 856 61 10029075

[B6] Sharma S Sharma MC Sarkar C Morphology of angiogenesis in human cancer: a conceptual overview, histoprognostic perspective and significance of neoangiogenesis Histopathology 2005 46 5 481 9 10.1111/j.1365-2559.2005.02142.x 15842629

[B7] Duff SE Li C Garland JM Kumar S CD105 is important for angiogenesis: evidence and potential applications FASEB J 2003 17 9 984 92 10.1096/fj.02-0634rev 12773481

[B8] Cheifetz S Bellón T Calés C Vera S Bernabeu C Massagué J Letarte M Endoglin is a component of the transforming growth factor-beta receptor system in human endothelial cells J Biol Chem 1992 267 27 19027 30 1326540

[B9] Goumans MJ Lebrin F Valdimarsdottir G Controlling the angiogenic switch: a balance between two distinct TGF-b receptor signaling pathways Trends Cardiovasc Med 2003 13 7 301 7 10.1016/S1050-1738(03)00142-7 14522471

[B10] Fonsatti E Altomonte M Nicotra MR Natali PG Maio M Endoglin (CD105): a powerful therapeutic target on tumor-associated angiogenetic blood vessels Oncogene 2003 22 42 6557 63 10.1038/sj.onc.1206813 14528280

[B11] Chien CY Su CY Hwang CF Chuang HC Chen CM Huang CC High expressions of CD105 and VEGF in early oral cancer predict potential cervical metastasis J Surg Oncol 2006 94 5 413 7 10.1002/jso.20546 16967447

[B12] Kyzas PA Agnantis NJ Stefanou D Endoglin (CD105) as a prognostic factor in head and neck squamous cell carcinoma Virchows Arch 2006 448 6 768 75 10.1007/s00428-006-0195-4 16612622

[B13] El-Naggar EK Huvos AG Barnes L, Eveson JW, Reichart P, Sidransky D Adenoid cystic carcinoma. I World Health Organization Classification of Tumours. Pathology and Genetics of Head and Neck Tumours 2005 Lyon: IARC Press 263 265

[B14] Vermeulen PB Gasparini G Fox SB Colpaert C Marson LP Gion M Beliën JA de Waal RM Van Marck E Magnani E Weidner N Harris AL Dirix LY Second international consensus on the methodology and criteria of evaluation of angiogenesis quantification in solid human tumours Eur J Cancer 2002 38 12 1564 79 10.1016/S0959-8049(02)00094-1 12142044

[B15] Singh S Sadanandam A Singh RK Chemokines in tumor angiogenesis and metastasis Cancer Metastasis Rev 2007 26 3-4 453 67 10.1007/s10555-007-9068-9 17828470 PMC4237067

[B16] Chen AM Garcia J Granchi PJ Johnson J Eisele DW Late recurrence from salivary gland cancer: when does "cure" mean cure Cancer 2008 112 2 340 4 10.1002/cncr.23165 18008358

[B17] Wal JE van der Becking AG Snow GB Waal I van der Distant metastases of adenoid cystic carcinoma of the salivary glands and the value of diagnostic examinations during follow-up Head Neck 2002 24 8 779 83 10.1002/hed.10126 12203804

[B18] Ellis GL Auclair PL Tumors of the salivary glands. Atlas of tumor pathology3rd series, fascicle 17 1996 Washington, DC: Armed Forces Institute of Pathology

[B19] Maruya S Shirasaki T Nagaki T Kakehata S Kurotaki H Mizukami H Shinkawa H Differential expression of topoisomerase IIα protein in salivary gland carcinomas: histogenetic and prognostic implications BMC Cancer 2009 9 72 19250538 10.1186/1471-2407-9-72 PMC2654461

[B20] Saad RS Jasnosz KM Tung MY Silverman JF Endoglin (CD105) expression in endometrial carcinoma Int J Gynecol Pathol 2003 22 3 248 53 10.1097/01.PGP.0000070852.25718.37 12819391

[B21] Minhajat R Mori D Yamasaki F Sugita Y Satoh T Tokunaga O Organ-specific endoglin (CD105) expression in the angiogenesis of human cancers Pathol Int 2006 56 12 717 23 10.1111/j.1440-1827.2006.02037.x 17096728

[B22] Ding S Li C Lin S Yang Y Liu D Han Y Zhang Y Li L Zhou L Kumar S Comparative evaluation of microvessel density determined by CD34 or CD105 in benign and malignant gastric lesions Hum Pathol 2006 37 7 861 6 10.1016/j.humpath.2006.02.006 16784986

[B23] Costa AF Demasi AP Bonfitto VL Bonfitto JF Furuse C Araújo VC Metze K Altemani A Angiogenesis in salivary carcinomas with and without myoepithelial differentiation Virchows Arch 2008 453 4 359 67 10.1007/s00428-008-0664-z 18795324

[B24] Zhang J Peng B In vitro angiogenesis and expression of nuclear factor kappaB and VEGF in high and low metastasis cell lines of salivary gland Adenoid Cystic Carcinoma BMC Cancer 2007 7 95 17543095 10.1186/1471-2407-7-95 PMC1903362

[B25] Barsky SH Karlin NJ Myoepithelial cells: autocrine and paracrine suppressors of breast cancer progression J Mammary Gland Biol Neoplasia 2005 10 3 249 60 10.1007/s10911-005-9585-5 16807804

[B26] Nguyen M Lee MC Wang JL Tomlinson JS Shao ZM Alpaugh ML Barsky SH The human myoepithelial cell displays a multifaceted anti-angiogenic phenotype Oncogene 2000 19 31 3449 59 10.1038/sj.onc.1203677 10918603

